# Cutaneous Metastases in Progressive Prostate Adenocarcinoma: A Case Report and Literature Review

**DOI:** 10.7759/cureus.46219

**Published:** 2023-09-29

**Authors:** Yesenia Brito, Bruce W Wilson, Kavonne I Bacchus, Janice Mwaniki, Juaquito Jorge, Frederick Tiesenga

**Affiliations:** 1 Surgery, St. George's University School of Medicine, True Blue, GRD; 2 Medicine, Richmond Gabriel University, Kingstown, VCT; 3 General and Bariatric Surgery, Tiesenga Surgical Associates, Elmwood Park, USA; 4 General Surgery, West Suburban Medical Center, Chicago, USA

**Keywords:** prostate-specific antigen (psa), adenocarcinoma, oncology, prostate cancer, cutaneous metastasis

## Abstract

Prostate cancer has an indolent progression course and commonly metastasizes to the vertebrae bone and regional lymph nodes. We report a patient with prostate cancer who has developed cutaneous metastases in multiple regions, including the right infraclavicular and abdominal area, as well as the left supraclavicular region. It presented as isolated, prominent nodules that were microscopically proven to be of prostate adenocarcinoma when biopsied. This rare presentation is a marker of an advanced disease course with a poor prognosis in castrate-resistant prostate cancer. Thorough clinical examination to rule out metastasis from the prostate and other dermatological conditions is paramount as well as ensuring early detection and optimizing patient outcomes.

## Introduction

Prostate cancer is the world’s second most commonly diagnosed malignant male tumor and the fifth leading cause of male cancer mortality [1,2]. In 2020, GLOBOCAN reported 1,414,259 new prostate cancer cases and 375,304 related deaths worldwide [2]. Some risk factors for prostate cancer include age, family history, genetic mutations observed with breast cancer 1 (BRCA1) and breast cancer 2 (BRCA2) genes, or mismatch repair gene mutations in Lynch syndrome [1,2]. Ethnicity also contributes to the risk of prostate cancer due to a reported higher incidence among black men [2].

Prostate cancer may present with symptoms similar to benign prostate hyperplasia (BPH) such as frequency, nocturia, poor or intermittent stream, hesitancy, straining to void, hematuria, terminal or post-micturition dribbling, urgency, and incomplete bladder emptying [1,3]. Additional symptoms, such as tingling, leg weakness, pain, paralysis, or fecal and urinary incontinence due to spinal cord compression, can be present on physical examination [3]. A hard or firm nodule on a digital rectal exam (DRE) is a typical physical finding of prostate cancer [3]. While the DRE, symptoms, and prostate-specific antigen (PSA) testing can support the diagnosis, the gold standard for diagnosis remains transrectal (TRUS) or transperineal (TPUS) ultrasound-guided prostate biopsies [1,3]. Prostate magnetic resonance imaging (MRI) is an advantageous diagnostic imaging technique that provides higher soft tissue resolution, accuracy, and reliability compared to TRUS or TPUS biopsies, especially when cancer is strongly suspected despite a negative initial biopsy [3]. MRI can also aid in surgical planning when radical prostatectomy is being considered [3].

Prostate cancer is often discovered in the peripheral zone of the prostate during the DRE [3]. It has a tendency to metastasize to the bone and lymph nodes, typically appearing as mixed osteolytic and osteoblastic lesions in areas like the vertebrae, hips, and pelvis [3,4]. Cutaneous metastases are rare, with a reported incidence of 0.36%, and are associated with poor prognosis [5]. Although the mechanism of metastases is not fully understood, it may occur through direct extension from another site of metastasis such as a subcutaneous lymph node, lymphatic or hematogenous spread, or a combination of these [6].

Clinically, skin metastases frequently imitate other dermatological conditions and often appear as infiltrated plaques or nodules [5] in the suprapubic region and anterior thigh [7]. Skin biopsies of these lesions are necessary for accurate diagnosis [5].

This case report presents a 64-year-old male with leuprolide-resistant prostatic adenocarcinoma and a decline in the ability to provide self-care who underwent an excisional biopsy of direct extension from another site of a clinically concerning cutaneous nodule.

## Case presentation

A 64-year-old Caucasian male of European descent presented to the emergency department (ED), reporting a two-week history of general weakness. The patient reported experiencing an unintentional weight loss of 20-25 pounds over the past several months, along with a one-week history of decreased appetite and inability to tolerate solids. Despite this, he continued to consume Ensure. Upon arrival in the ED, he was covered in urine and feces. He admitted to a major decline in his ability to care for himself.

The patient's past medical history is significant for metastatic prostate adenocarcinoma cancer (PAC). He was diagnosed at the age of 63 with a stage 4 PAC. At that time, he presented with foot pain and lymphadenopathy. His genomic studies were positive for the CHECK2 heterozygous mutation involving the deletion of exon 8-9. The prostate biopsy was confirmed as adenocarcinoma, involving three of three cores and 80% of the total tissue. The Gleason grade was 4+5 (score=9)(grade group 5), confirmed by PIN4 IHC-G. The patient was started on hormonal therapy and is compliant with treatment. He is a current smoker with a history of one pack for 25 years. The patient’s family history is positive for endometrial and uterine cancer in the mother, who was diagnosed at the age of 73.

**Figure 1:**
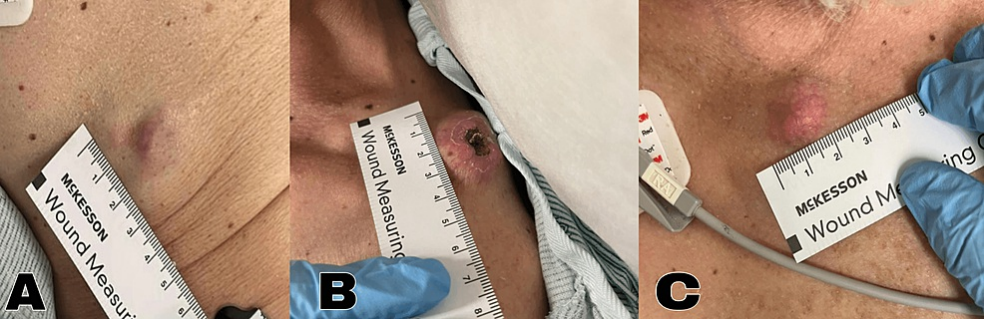
(A) Painless mobile right abdominal mass, measuring 3x3 cm; (B) Painless mobile left supraclavicular mass measuring 3x4 cm exhibiting a black central eschar and telangiectasia; (C). Painless mobile right infraclavicular mass measuring 2x2 cm, exhibiting telangiectasia

Upon assessment, the patient presented with a slightly elevated temperature of 100.6 F (38.1 C), along with tachycardia with a pulse of 115 beats per minute (BPM) and tachypnea with a respiratory rate of 28 BPM. The patient's blood pressure was measured at 154/74 mmHg, and his weight was 120 pounds, resulting in a body mass index (BMI) of 18.79 kg/m^2^. Oxygen saturation levels were within normal limits, measuring at 98% on room air. The patient was observed to be alert, cooperative, and cachectic. He exhibited erythema over the sacrum and gluteal region. He also had a painless mobile abdominal mass measuring 3x3 cm (Figure 1A). Additionally, he had another painless and mobile mass on the left shoulder (Figure 1B) and right infraclavicular region (Figure 1C) measuring 3x4 cm and 2x2, respectively. The left shoulder mass showed central eschar telangiectasias, whereas the right infraclavicular mass displayed telangiectasia.

The patient's laboratory tests revealed significant abnormalities, including suspected sepsis and pyelonephritis. The complete blood count (CBC), prothrombin time (PT), and international normalized ratio (INR) established no anemia, lymphoma, or hepatic dysfunction. The prostate-specific antigen (PSA) showed elevated levels (Table 1).

**Table 1:** Laboratory parameters k/mm³ = thousand per cubic millimeter; m/mm³ = million per cubic millimeter; g/dL = grams per deciliter; mg/dL= milligrams per deciliter; mmol/L = millimoles per liter; mL/min = milliliters per minute; m² = square meter; sec = second; IU/L = international units per liter; hpf = high-power field

Test Name	Result	Reference Range
CBC		
White Blood Cell Count	21.2 k/mm³	4.5 - 11.0 k/mm³
Neutrophil Percentage	89.10%	40 - 74%
Absolute Neutrophil Count	18.9 k/mm³	2.0 - 7.5 k/mm³
Red Blood Cell Count	4.11 m/mm³	4.5 - 5.5 m/mm³
Hemoglobin	12.2 g/dL	13.5 - 17.5 g/dL
Hematocrit	35.30%	38.8 - 50.0%
Platelet Count	819 k/mm³	150 - 400 k/mm³
PT/INR		
Prothrombin Time	14.6 sec	10.3 - 12.4 sec
INR	1.3	0.9 - 1.1
PTT	32 sec	23 - 33 sec
PSA		
06/08/2022	906 ng/mL	
12/15/2022	458 ng/mL	
03/08/2023	1075 ng/mL

**Figure 2:**
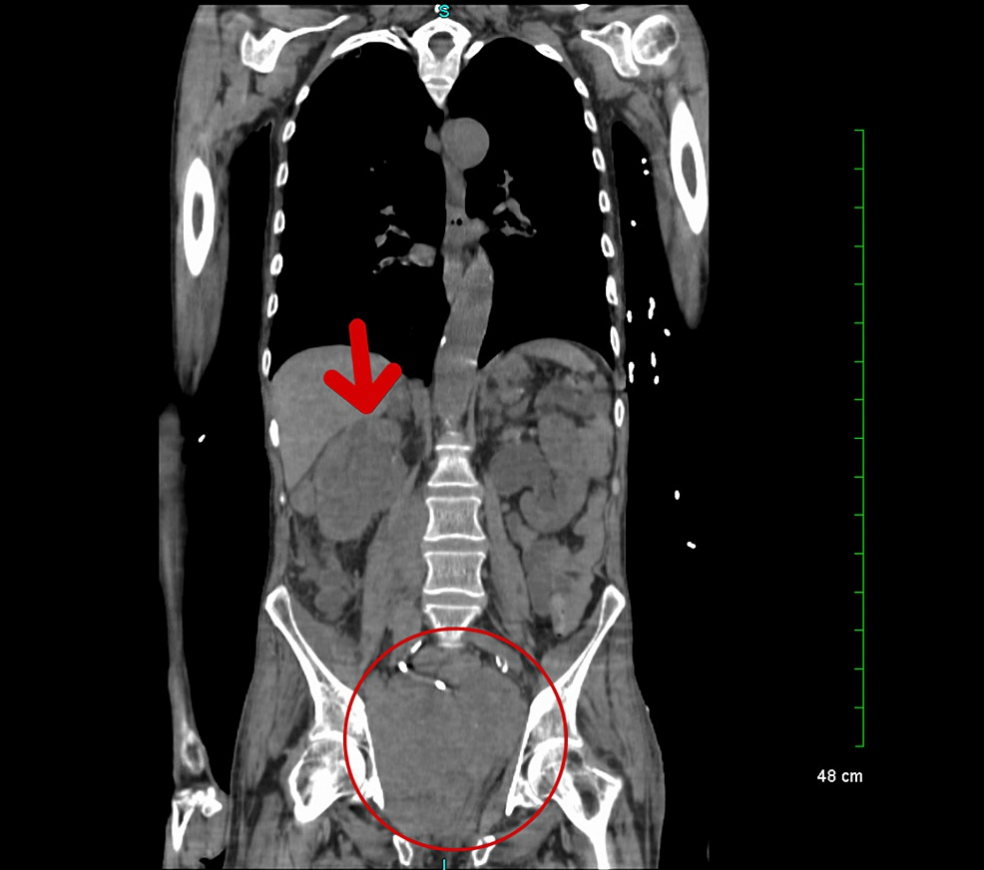
Progression of the spread of the malignant disease

**Figure 3:**
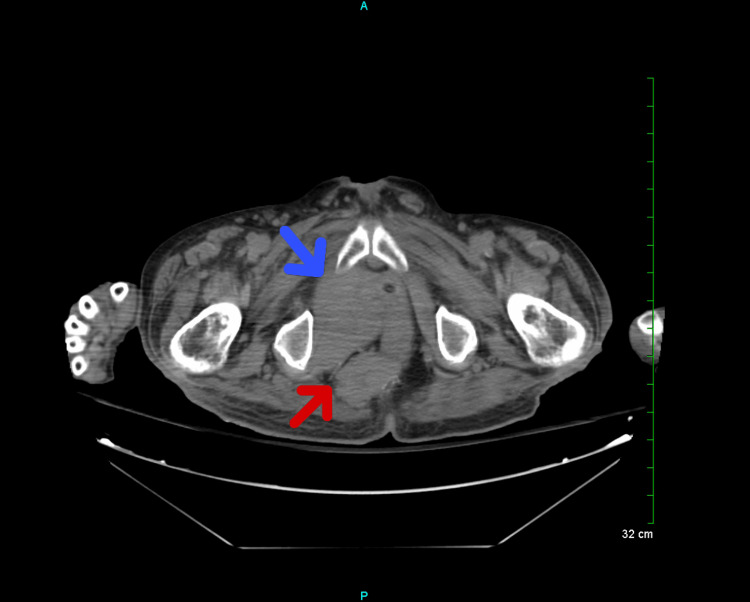
Large bulky pelvic mass measuring 12.0 x 12.0 cm in the greatest anterior-posterior by transverse dimensions by 13 cm in the greatest craniocaudad dimension (blue arrow). Mass in the ischiorectal fossa posteriorly measured 5.4 x 3.6 cm (red arrow). cm = centimeter

**Figure 4:**
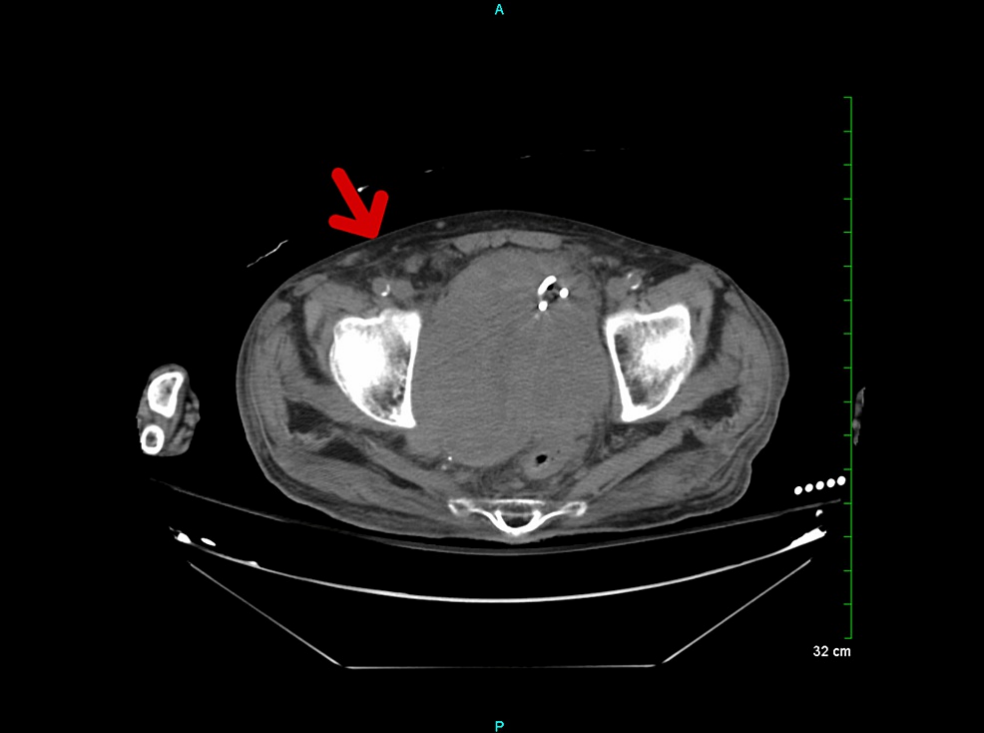
Soft tissue nodule on the right abdominal wall

A computed tomography (CT) of the chest, abdomen, and pelvis without contrast showed the progression of widespread malignant disease (Figure 2). An interval increase in the size of a large conglomerate mass in the right hemipelvis, with an increased mass effect on the urinary bladder (Figure 3), was also noted. Additionally, a soft tissue nodule on the right abdominal wall has increased in size from the prior (Figure 4). Prominent lower left axillary lymph nodes were also noted.

The patient's history and physical condition were concerning for uncontrolled metastatic prostate cancer. The surgical team was consulted for a biopsy of metastatic nodules, as there was concern for other primary sources.

**Figure 5:**
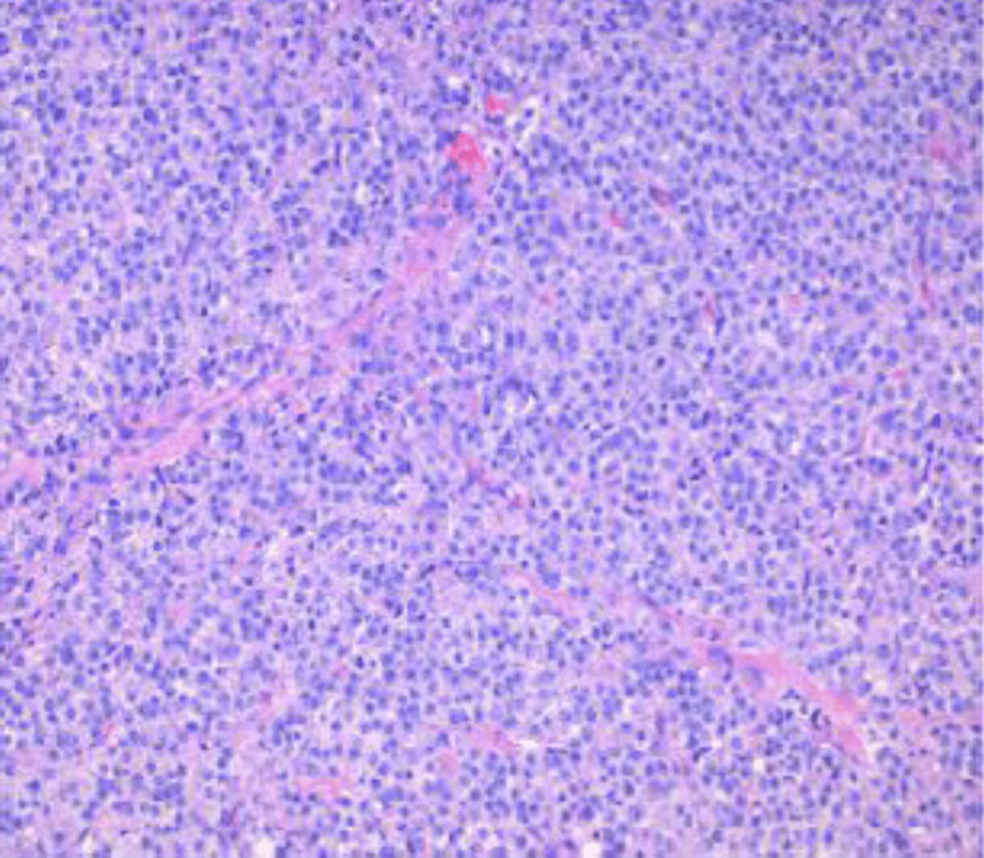
High-grade metastatic prostatic adenocarcinoma Tumor cells positive for AE1/AE3, PSA, and NKX3.1. The tissue tested negative for thyroid transcription factor (TTF-1), Synaptophysin, S-100, keratin 20 (CK20), and CD20.

In the operating room (OR), the surgical team excised an approximately 3x4 cm mass from the right chest. The mass was sent for pathological evaluation. The report showed tumor cells positive for pan-keratin cocktail immunohistochemical stain AE1/AE3, prostate-specific antigen (PSA), and NKX3.1 homeobox protein (Figure 5). These findings support the diagnosis of metastatic prostatic adenocarcinoma.

The patient continued to receive inpatient treatment to improve his quality of life, given the significant progression of metastatic cancer. After completion of inpatient treatment, the patient's ongoing care will be managed by a range of specialists with a focus on palliative care in a hospice facility.

## Discussion

Prostatic carcinomas (PC), and specifically its most common manifestation of a prostatic (acinar) adenocarcinoma (PAC), arise from a malignant transformation of the cells that produce prostatic fluid [8].

This type represents almost all encountered cancers of the prostate [8]. Other types include small cell carcinomas, neuroendocrine tumors, urothelial cell carcinomas, and sarcomas [1]. Age is the most clear risk factor for prostate cancer [8]. PC is usually asymptomatic in the early stages and commonly presents as benign prostatic hyperplasia when it becomes symptomatic [9].

While prostate cancer is rare in men below the age of 40, the probability of developing the disease rises after the age of 50 [10]. Approximately 60% of cases are seen in patients over the age of 65 [10]. Black Americans and Caribbeans have a higher incidence of prostate cancer, whereas Asians, Hispanics, and Native Americans have a lower occurrence, and Caucasians fall between the two groups [1,2].

There are various genetic mutations that are known to increase the risk of prostate cancer, such as BRACA1 and BRACA2, as well as hereditary non-polyposis colorectal cancer (Lynch syndrome) [10]. Additionally, family history plays a significant role in determining risk; having a brother or father with prostate cancer doubles a patient's risk, and having more than one relative increases it further [10]. However, it's worth noting that most cases are sporadic [10]. Other potential risk factors include diet (such as high calcium and dairy product consumption), obesity, smoking, chemical exposure (for example, through occupational exposure in firefighters or exposure to Agent Orange), prostatitis, sexually transmitted infections (STIs), and vasectomy [1].

PC is the second leading cause of cancer death among men. In 2020, lung/bronchus cancer was responsible for 72,949 deaths, PC 32,707 deaths, and colon/rectal cancer 28,043 deaths [11]. However, PCs are more common than either of these. In 2019, 224,733 cases of PC were reported in the US while 221,097 and 142,462 cases were reported of lung/bronchus and colon/rectum cancer, respectively [12]. This is reflected in the five-year survival rate [13]. For localized and regional cancers, the five-year survival rate is over 99%, with the combined rate of all Surveillance, Epidemiology, and End Results (SEER) stages being 97% [13]. The survival rate drops to 32% in cases diagnosed with distant metastases [3].

PC treatment options vary based on the severity of the disease. For low-grade tumors that grow slowly, treatment may not be necessary [3]. However, patients with prostate cancer who require intervention have several options available, including periodic PSA testing, prostate MRI, radiation therapy, focal ablative treatment, and pharmacotherapy [3]. Luteinizing hormone-releasing hormone (LHRH) agonists like leuprolide and goserelin, as well as anti-androgen therapy like bicalutamide, can be used for patients with a PSA level above 10 ng/ml [3]. Injectable degarelix or oral relugolix can also be used to cause a rapid decline in testosterone levels without a surge [3]. However, treatment is complicated by unclear indications for PC PSA screening, which has been associated with overdiagnosis rates between 20% and 30% [4]. A 13-year study showed that only 4.3 men per thousand were able to avoid death/metastasis due to screening [4]. Serious complications due to treatment were seen in 70 men per thousand [4]. Due to these findings, routine PSA screening is not recommended by the US Preventive Service Task Force [4]. These changes in PSA screening recommendations began changing in 2008. This has resulted in a reduction in the incidence of local and regional PC by 37% [14]. However, there has been an increase in the diagnosis of metastatic PC of 72% from 2007 to 2013 [5]. Prostate cancer most commonly spreads to the adrenal glands, bone, liver, and lungs. PC metastasizes to other organs more rarely. At initial presentation, 77% of cases are local, 13% have spread to the regional lymph nodes, and 6% are metastatic [5].

It is evident that our patient has an aggressive form of prostate cancer. He initially presented with stage M1C (tumor, node, metastasis (TNM) classification) in June 2022 and was subsequently put on hormonal therapy. At that time, he had the ability to ambulate and take care of himself. However, by March 2023, he had become bedridden and was unable to perform even the most basic self-care tasks. As a result, he was placed under hospice care at the end of his hospital admission. This case highlights the potential benefits of earlier and more aggressive treatment for patients like him. Cases like this are important to study. By identifying genetic markers that predispose patients to aggressive prostate cancer, we could implement targeted screening for this population. Such an approach would enable the earlier initiation of more aggressive treatments, with the potential for significant benefits. Routine screening of these high-risk patients would likely outweigh the associated costs, unlike those who are likely to develop a less aggressive form of the disease. This would resemble the increased colon cancer screening performed on individuals with genetic predispositions to colorectal cancer.

Upon the patient's presentation at the ED in March 2023, it was observed that he had three metastatic skin nodules. These nodules were located in the left supraclavicular, right infraclavicular, and right abdominal regions. This particular finding is exceedingly rare, occurring in only 0.36% of prostate cancer cases, and is known to be associated with a poor prognosis, as demonstrated in this patient [7]. It is crucial to perform a biopsy on these lesions since they can resemble other skin conditions such as infiltrated plaques or nodules [7].

In this case, the lesions strongly resembled basal cell carcinoma, exhibiting characteristics like telangiectasia and central eschar. Immunostaining of the right infraclavicular nodule revealed positive results for pan-keratin cocktail immunohistochemical stain AE1/AE3, PSA, and NKX3.1, which are tissue markers typically observed in prostatic adenocarcinoma. Conversely, the tumor cells were negative for thyroid transcription factor (TTF-1), Synaptophysin, S-100, keratin 20 (CK20), and CD20. These pathological findings strongly support the diagnosis of metastatic prostate cancer. This case underscores the critical role of performing a biopsy and relying on pathology results. Had it been assumed that the lesions were skin cancer based on their appearance, an important diagnosis could have been missed in a patient presenting with similar cutaneous nodules, but otherwise asymptomatic.

## Conclusions

This case report emphasizes the rare occurrence of distant cutaneous metastases in a patient with prostate cancer and a declining ability to provide self-care. Palliative care was initiated due to advanced cancer and comorbidities. The diagnostic challenges arise from the atypical presentation of metastases, resembling other dermatological conditions, requiring awareness of prostate cancer status and immunohistochemistry. The role of pharmacotherapy resistance, like leuprolide, in this phenomenon remains uncertain.
